# Compression of the Lateral Antebrachial Cutaneous Nerve due to Leakage of Iron after an Intravenous Iron Infusion

**DOI:** 10.3390/diagnostics10080516

**Published:** 2020-07-25

**Authors:** Soyoung Kwak, Min Cheol Chang

**Affiliations:** Department of Physical Medicine and Rehabilitation, College of Medicine, Yeungnam University, Daegu 42415, Korea; sk315@ynu.ac.kr

**Keywords:** intravenous iron infusion, staining, complication, cutaneous nerve, entrapment, ultrasonography

## Abstract

Skin staining due to iron leakage into the subcutaneous tissue can sometimes occur during intravenous iron infusion. We describe a case of lateral antebrachial cutaneous nerve (LACN) entrapment due to extravasated iron after an intravenous iron infusion. A 41-year-old woman received an intravenous ferric carboxymaltose infusion for iron deficiency anemia. However, during the infusion, extravasation of iron occurred and brown pigmentation developed on the lateral side of the cubital fossa. Sixteen months later, the patient still had some staining in her anterolateral elbow and proximal forearm. In addition, she complained of tingling pain over her left forearm. Ultrasonography (US) revealed a lateral antebrachial cutaneous nerve (LACN) under the stained area. When we swept the stained area with the US transducer, she reported a tingling pain on her left lateral forearm, the region innervated by the left LACN. Therefore, we considered that the pain resulted from the compression of the left LACN by the leaked iron during the intravenous infusion. Leaked iron can compress the cutaneous nerve and result in neuropathic pain and cosmetic problems. When patients with skin staining after iron infusion have neuropathic pain, clinicians should consider the possibility of entrapment of the cutaneous nerves.

**Figure 1:**
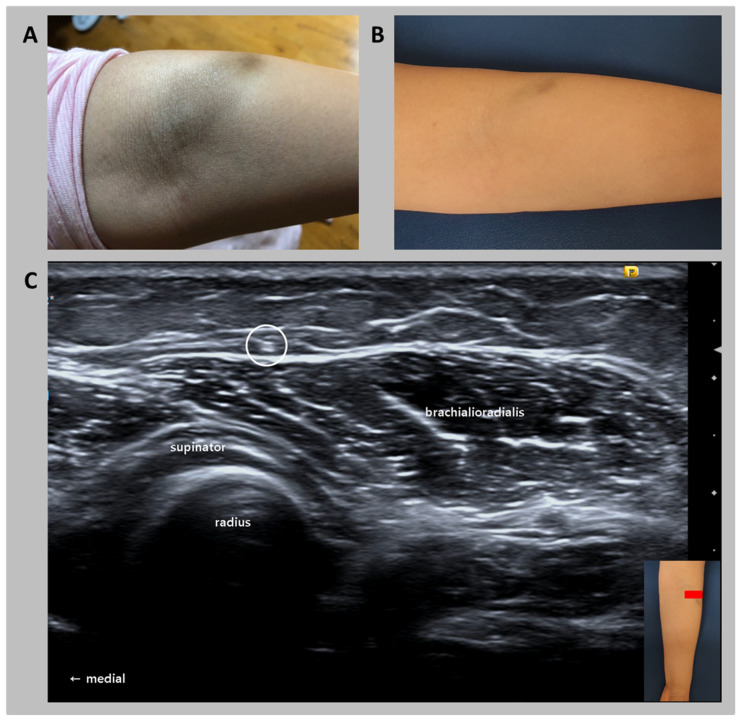
(**A**) One day after the intravenous ferric carboxymaltose infusion. The area around the left cubital fossa was largely stained by the extravasated iron. (**B**) Sixteen months after the infusion, the staining had faded remarkably but with some staining remaining. (**C**) The ultrasonography image shows a lateral antebrachial cutaneous nerve (in the circle) under the stained area in the anterolateral elbow and proximal forearm. A 41-year-old woman presented with tingling pain over her left forearm. Around 16 months before, the patient received an intravenous ferric carboxymaltose infusion for iron deficiency anemia. Within minutes of commencing the infusion, pain around the cannula insertion site occurred. The infusion was stopped, and the nurse drew out the cannula. By the following day, a painful brown pigmentation, 4.0 × 8.5 cm in size, had developed on the lateral side of the cubital fossa around the cannula insertion site (Figure 1A). After 16 months, a large part of the staining had fade, with 2.0- × 4.3-cm staining remaining (Figure 1B). In addition, when the stained area was compressed by an external force, the patient felt a tingling pain on her left lateral forearm. Motor and sensory deficits were not observed. Ultrasonography (US) imaging (18-MHz linear transducer; S2000, Siemens, Seoul, South Korea) could not visualize the remaining iron in the subcutaneous tissue. However, when the stained area was swept with the transducer, the patient described a tingling pain on her left lateral forearm, equal to her usual pain (Figure 1C). US imaging revealed a lateral antebrachial cutaneous nerve (LACN) under the stained area, and the painful area at the forearm where the US transducer passed over was consistent with the region innervated by the left LACN. Accordingly, the patient’s pain seemed to have resulted from the compression of the left LACN by the leaked iron during the intravenous infusion. Nevertheless, the patient perceived pain only when the transducer was swept over the stained area on her left arm. Therefore, although the remaining leaked iron was above the LACN, the nerve did not have direct contact with the iron. Written informed consent was obtained from the patients for publication of this case report. The study was approved by the local Institutional Review Board of our hospital. In patients with iron deficiency anemia, intravenous iron infusion is being widely applied owing to its advantage of increasing hemoglobin levels and body iron stores quickly and effectively [1]. Intravenous iron infusion was demonstrated to be a safe treatment method with few side effects [2,3]. However, some previous studies reported the occurrence of skin staining due to iron leakage into the subcutaneous tissue [4,5]. All the patients in those studies did not complain of pain. However, cutaneous nerves pass through the subcutaneous tissue; therefore, we can infer that the iron that leaked into the subcutaneous tissue can compress the cutaneous nerves like in our case [6]. In our case, the diagnosis of LACN entrapment was made straightforward after observing the proximity of the LACN to the stained area. Furthermore, dynamic compression with the transducer was conducted to confirm the LACN entrapment. Nerve conduction studies are known to be the gold standard for diagnosing nerve entrapment. However, because of the wide anatomical variations and unpredictable anastomosis of cutaneous nerves, results of nerve conduction studies can be inaccurate. US imaging is helpful for the diagnosis of pathologies of the cutaneous nerves [7]. For the evaluation of the LANC at the elbow and proximal forearm level, the transducer is placed on the elbow crease. Then, the LACN can be found lateral to the biceps tendon, and the cephalic vein is located beside the LACN [7]. It passes distally over the brachioradialis muscle. After iron infusion, skin staining is known to occur in approximately 1.6% of patients [8]. Leaked iron can compress the cutaneous nerve and result in neuropathic pain and cosmetic problems. Therefore, skin staining due to extravasation of iron into the subcutaneous tissue is an important side effect of intravenous iron infusion. To avoid this side effect, clinicians or nurses should be aware of and monitor for the possible occurrence of iron extravasation. Moreover, when patients with skin staining after iron infusion complain of neuropathic pain, clinicians should consider the possibility of entrapment of the cutaneous nerves. US imaging would be useful for diagnosing pathologies of the cutaneous nerves.

## References

[1] Koch T.A., Myers J., Goodnough L.T. (2015). Intravenous Iron Therapy in Patients with Iron Deficiency Anemia: Dosing Considerations. Anemia.

[2] Govindappagari S., Burwick R.M. (2019). Treatment of Iron Deficiency Anemia in Pregnancy with Intravenous versus Oral Iron: Systematic Review and Meta-Analysis. Am. J. Perinatol..

[3] Holm C., Thomsen L.L., Norgaard A., Langhoff-Roos J. (2015). Intravenous iron isomaltoside 1000 administered by high single-dose infusions or standard medical care for the treatment of fatigue in women after postpartum haemorrhage: Study protocol for a randomised controlled trial. Trials.

[4] Crowley C.M., McMahon G., Desmond J., Imcha M. (2019). Skin staining following intravenous iron infusion. BMJ Case Rep..

[5] Thompson J., Pavord S., Lim K. (2014). Severe haemosiderin pigmentation after intravenous iron infusion. Intern Med J..

[6] Chang M.C., Chang K.V., Wu W.T., Özçakar L. (2020). Ultrasound Imaging for Painful Lipomatosis: Cutaneous Nerves Really Matter!. Am. J. Phys. Med. Rehabil..

[7] Chang K.V., Mezian K., Naňka O., Wu W.T., Lou Y.M., Wang J.C., Martinoli C., Özçakar L. (2018). Ultrasound Imaging for the Cutaneous Nerves of the Extremities and Relevant Entrapment Syndromes: From Anatomy to Clinical Implications. J. Clin. Med..

[8] Baird-Gunning J., Bromley J. (2016). Correcting iron deficiency. Aust. Prescr..

